# Prenatal Exposure to Heavy Metals Affects Gestational Age by Altering DNA Methylation Patterns

**DOI:** 10.3390/nano11112871

**Published:** 2021-10-28

**Authors:** Eun Jung Koh, So Yeon Yu, Seung Hwan Kim, Ji Su Lee, Seung Yong Hwang

**Affiliations:** 1 Department of Bio-Nanotechnology, Hanyang University, Sangnok-gu, Ansan 15588, Korea; koeunjung22@naver.com (E.J.K.); kandoli1@daum.net (S.H.K.); 2 Department of Molecular & Life Science, Hanyang University, Sangnok-gu, Ansan 15588, Korea; yusso3027@naver.com (S.Y.Y.); jisu5487@gmail.com (J.S.L.); 3 Department of Applied Artificial Intelligence, Hanyang University, Sangnok-gu, Ansan 15588, Korea

**Keywords:** heavy metals, preterm birth, DNA methylation, prenatal exposome

## Abstract

Environmental exposure is known to have toxic effects. Maternal environmental exposure not only affects mothers but also their fetuses in utero, which may interrupt their early development. Preterm birth, one of the outcomes of prenatal exposure, is a significant factor in lifelong health risks. To understand the effects of prenatal exposome on preterm birth, we studied the association between maternal and prenatal heavy metal exposure and gestational age, using resources from the MOthers’ and Children’s Environmental Health (MOCEH) study in South Korea. Additionally, a methylation assay was performed to analyze epigenetic mediation using genomic DNA derived from the cord blood of 384 participants in the MOCEH study. The results suggest that maternal cadmium exposure is associated with a decrease in gestational age through an alteration in DNA methylation at a specific CpG site, cg21010642. The CpG site was annotated to a gene involved in early embryonic development. Therefore, irregular methylation patterns at this site may contribute to premature birth by mediating irregular biological mechanisms.

## 1. Introduction

The exposome is defined as the totality of exposure during one’s lifetime [1] and consists of three factors: the external environment, the internal environment, and health risks. Exposome research aims at understanding these factors systemically—how environmental exposure to various external sources alters one’s biomolecular regulation systems and how these alterations lead to health problems. However, even though the concept appeared more than a decade ago and some studies have focused on the integrated exposome concept [2], much research in this area is fragmentary and only considers individual associations between three factors of the exposome: (1) environmental exposure and health risks; (2) environmental exposure and epigenomics; and (3) health risks and epigenomics. Pregnant women and their fetuses are noteworthy targets of exposome studies. According to the developmental origins of health and disease (DOHaD) theory, maternal environmental exposure is easily transported to the fetus and could affect early developmental programming, which may result in health problems during the lifetime of the offspring [3].

Heavy metals, such as mercury (Hg), lead (Pb), and cadmium (Cd), are representative environmental toxicants. Humans are exposed to heavy metals through various pathways in their normal lives. For example, Hg exposure can occur through fish intake, dental amalgam, thermometer use, or skin creams; Pb exposure occurs through paints used in households or toys and ground water contaminated by Pb-containing plumbing; and Cd exposure occurs through drinking water contaminated by soil pollution, industrial activity, waste combustion, or smoking [4,5,6,7]. Because of their low excretion efficiency, heavy metals accumulate in the body and exert neuro- or immunotoxic effects during early development, resulting in abnormal cellular mechanisms [8]. Cd forms a protein–ligand complex with metallothionein in the placental tissue, which inhibits Cd transmission from mother to fetus [9].

DNA methylation refers to the addition of a methyl group (–CH_3_) to the fifth carbon of a cytosine followed by a guanine, which is termed as the CpG site or CpG dinucleotide. Usually, DNA methylation occurs in CpG islands where CpG sites are arranged repetitively. Interestingly, approximately 70% of promoter regions are located near CpG islands [10]; therefore, DNA methylation has a close inverse relationship with gene expression. High levels of DNA methylation (hyper-methylation) repress gene expression by lowering the accessibility of transcription factors through alterations in the DNA structure or through the recruitment of proteins involved in repression, with an opposite effect occurring in hypo-methylation. One notable feature of DNA methylation is that its regulation is vulnerable to external factors, such as environmental exposure [11]. According to the central dogma, irregular epigenetic regulation successively affects gene expression, protein coding, and phenotypes with unfavorable outcomes.

The ultimate outcome of environmental exposure is chronic health problems or disease occurrence. Preterm birth (PTB), which is a birth taking place before 37 weeks of gestation, or shortened gestational age (SGA), are regarded as some of the outcomes of prenatal environmental exposure. According to the World Health Organization (WHO), 15 million babies—accounting for 1% of total births worldwide—are born early every year [12]. The population and housing census in Korea also revealed that 8.5% of total births in 2020 were PTBs [13], and this proportion has consistently increased over the last decade. The main causes of PTB are intrauterine inflammation [14] and oxidative stress [15]. Some studies have considered heavy metals as another cause of PTB and determined the causal effects of heavy metal exposure on intrauterine inflammation and oxidative stress [16]. As PTB indicates incomplete in utero development, this immaturity may play a role as a foundational factor in experiencing lifelong health effects. For example, premature babies have a higher risk of retardation of neuronal development [17].

In this study, we aimed to prove the hypothesis that maternal heavy metal exposure causes alterations in DNA methylation patterns in the fetus and accounts for the possibility of SGA. We utilized the MOthers’ and Children’s Environmental Health (MOCEH) study, a large-scale birth cohort in South Korea. We compared the heavy metal levels measured in both maternal and cord blood and completed an assessment of the association between exposure and SGA. The DNA methylation patterns in cord blood were assayed and analyzed as mediating mechanisms between heavy metal exposure and SGA. This study reveals a serial association of factors that can help us understand the prenatal exposome.

## 2. Materials and Methods

### 2.1. Sample Preparation

The MOCEH study is one of the birth cohorts in South Korea that was established to help understand the effect of mothers’ environmental exposure on children’s health. The study recruited 1751 pregnant women from 2006 to 2010 [18], and all participants provided informed consent. Among the overall enrollment, we obtained 384 genomic DNAs (gDNAs) derived from cord blood and metadata, including blood heavy metal levels, birth outcomes, and several variables for data adjustment. The detailed method used for measuring heavy metal levels in blood was described in a previous study [18]. The samples and information were provided by the National Institute of Environmental Research (NIER) in Korea, which manages the resources of the MOCEH study. This study was approved by the Institutional Review Board of NIER (IRB No. NIER-2018-BR-010-01).

### 2.2. DNA Methylation Assay

One microgram of gDNA was subjected to bisulfite conversion using the EZ-96 DNA Methylation Kit (Zymo Research, CA, USA). Bisulfite-converted DNA was sequentially amplified, fragmented, and purified using an Infinium MethylationEPIC Kit (Illumina, CA, USA). The prepared DNA fragments were loaded onto a HumanMethylationEPIC array chip on which over 850 K probes were planted. The intensity of fluorescence emitted by the hybridization of a DNA sample and probes was imaged using the iScan System (Illumina) and yielded raw data in the ‘.idat’ format.

### 2.3. Data Quality Control

Prior to downstream analysis, 384 EPIC array data were preprocessed using the proper QC criteria [19]. First, samples with blank metadata were excluded from the analysis [20]. Other sample QC criteria were as follows: (1) poor bisulfite conversion rate (<0.85); (2) having more than one probe with a higher detection *p*-value (>0.001); and (3) gender discrepancy between metadata and the one predicted by specific probe intensities. Likewise, probes that corresponded to the following criteria were removed: (1) higher detection *p*-value (>10^–16^) in more than 5% of the total samples; (2) having cross-reactivity; and (3) annotated to a single nucleotide polymorphism (SNP) or sex chromosome.

Filtered data were converted into β values, which indicate methylation levels on a scale from 0 to 1. Probes, whose β range throughout the samples is less than 0.05, are regarded to be insensitive to external stimulation and were removed. Finally, the filtered β values were converted into M-values. which represent log_2_-transformed ratio of the intensities of methylated and unmethylated probes for a more intuitive analysis.

### 2.4. Data Adjustment

The blood cell type composition of each gDNA was obtained using a calculation algorithm [21] that uses specific probe intensities from the data, considering that heterogeneous cell types in blood have different DNA methylation patterns and may have confounding effects in downstream analyses. We used the cord blood reference panel [22] to include nucleated red blood cells (nRBCs) along with six common cell types (CD8+ T, CD4+ T, natural killer cell, B cell, monocyte, and granulocyte) in the calculation because nRBCs exist in cord blood but not in adult blood. Several confounding variables, such as infant sex, maternal age, BMI, parity, smoking history, batch effects from multiple experiments, and cell type composition were used for the adjustment of the DNA methylation data.

### 2.5. Bioinformatics and Statistical Analyses

The preprocessing of the EPIC array data and downstream analyses, including epigenome-wide association studies (EWAS) and statistical assessments, were performed in the R (version 4.0.4) environment using several packages, such as missForest (ver 1.4) [23], minfi (ver 1.36.0) [24], wateRmelon (ver 1.34.0) [25], maxprobes (ver 0.0.1) [26], sva (ver 3.38.0) [27], limma (ver 3.46.0) [28], DMRcate (ver 2.4.1) [29], and mediation (ver 4.5.0) [30]. Differentially methylated positions (DMPs) were defined by statistical cutoffs of *p* < 10^−8^ and *q* < 10^−5^.

## 3. Results

### 3.1. Sample and Data Preparation

A total of 8 out of 384 subjects were removed, because there was a complete lack of metadata for four, and the other four had outliers in data for birth weight, gestational age (GA), maternal Pb level, and prenatal Cd level. Missing values in the metadata of the remaining 376 subjects were imputed. In total, 9 samples and 242,378 probes were removed by preprocessing the methylation data. As a result, 367 samples and 623,481 probes were prepared for downstream analyses. The characteristics of the 367 samples are summarized in Table 1.

### 3.2. Assessment of Exposure

**Figure 1:**
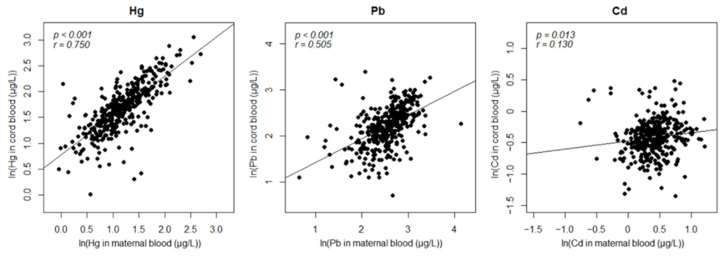
Association between maternal and prenatal heavy metal exposure levels.

Maternal and prenatal heavy metal levels were natural log-transformed to achieve a normal distribution. The association between heavy metal levels in mothers and infants and between the exposures and GA were assessed using a linear regression model. Maternal and prenatal exposures to Hg and Pb showed a strong positive association (Figure 1, Table 2), with Pearson’s correlation coefficients (r) of 0.750 and 0.505, respectively, whereas Cd had a negligible positive correlation (r = 0.130). Second, GA appeared to be positively correlated with Hg levels and negatively correlated with the Pb and Cd levels in both mothers and infants; however, the correlations were not statistically significant (Table 2).

### 3.3. EWAS on Heavy Metal Exposures and Birth Outcomes

**Figure 2:**
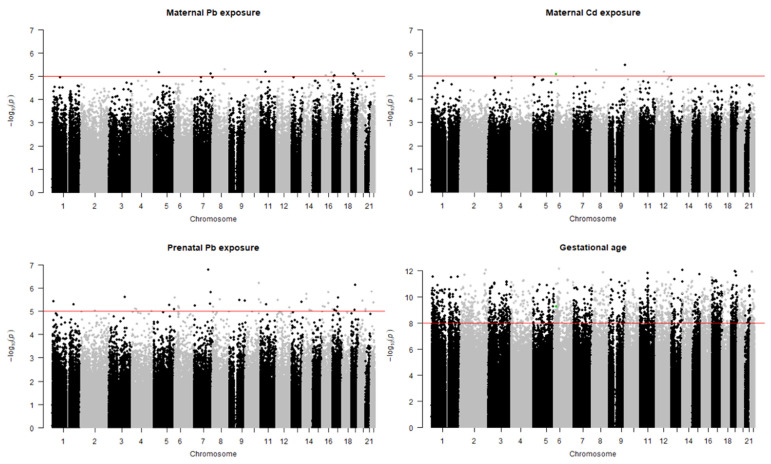
Manhattan plots for differentially methylated positions according to gestational age, birth weight, maternal Pb and Cd, and prenatal Pb exposure in order. Green dots in both gestational age and maternal Cd exposure indicate cg21010642.

Originally, 367 subjects were intended to be categorized by their exposure levels and GA for the case–control study. However, the exposure levels and GA were used as continuous variables in the analyses, as the data for the majority of subjects were skewed toward normal ranges. In EWAS, no differential methylation patterns according to exposure were observed. By lowering the cutoff to *p* < 10^−5^, 11, 4, and 46 differentially methylated positions (DMPs) were identified for maternal Pb and Cd and prenatal Pb exposure, respectively. In contrast, 2002 CpG sites were differentially methylated according to GA (*p* < 10^−8^, *q* < 10^−5^, Figure 2). The identified DMPs had quantitative associations with comparative factors rather than having on-off effects, as they were analyzed as continuous variables.

**Figure 3:**
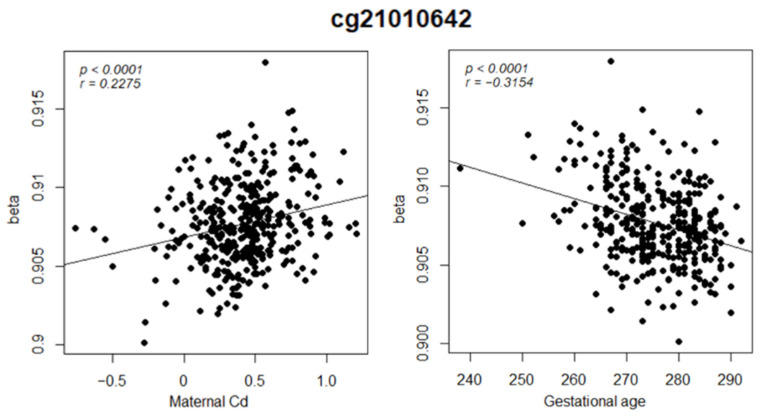
Differentially methylated patterns of cg21010642 according to maternal Cd exposure levels (**left**) and gestational age (**right**).

Interestingly, the methylation pattern of cg21010642 was simultaneously associated with both maternal Cd levels and GA. The methylation of cg21010642 was positively associated with maternal Cd exposure but inversely associated with GA (Figure 3, Table 3). To summarize, increased maternal Cd exposure may cause the hypermethylation of cg21010642, which leads to SGA. This particular association between maternal Cd levels and GA corresponded with the results of the linear regression model. Moreover, it was further proven that cg21010642 methylation has causal mediation effects between maternal Cd exposure and GA, with a mediation strength of −2.104 (95% CI: −3.271 to −1.09, *p* < 2 × 10^−16^), as determined by mediation analysis. Specific DNA methylation patterns mediate the relationship between exposure and health risks.

## 4. Discussion

Almost a decade ago in South Korea, many people were exposed to toxicants in humidifier disinfectants that were commonly used in households. The majority of the affected individuals were pregnant women and infants [31] who suffered from lifelong pulmonary disorders or even lost their lives. Therefore, it is no exaggeration to say that almost every living environment can be a source of exposure [32]. As the consequences of exposure are not instant and not immediately felt, environmental exposure in living surroundings is easily overlooked. However, toxicants gradually permeate the lives and influence the health of individuals. Therefore, it is essential to recognize and assess the environmental exposure risks, and exposome approaches should facilitate these interpretations.

In this study, the prenatal exposome concept was employed to study heavy metal exposure and its possible contribution to PTB. First, we discovered that maternal Hg or Pb exposure tends to be transmitted to the fetus. The slopes of the regression models for Hg and Pb were 0.92 and 0.78, respectively. Despite the fact that maternal exposure levels are gradually diminished by metabolism or half-lives, the quantity of exposure that fetuses receive is equivalent to that of the mother, and it persists throughout the time in utero. This might be because the body surface area of the fetus is much lower than that of the mother, which further implies that the magnitude of toxicity would be much stronger in the fetus.

Second, Cd appeared to have a negligible transmission rate. Nevertheless, the effects of small amounts of Cd toxicity have been consistently reported to influence health risks, including birth outcomes [33]. In this study, it was revealed that maternal Cd exposure is associated with SGA, which indicates a higher possibility of PTB. Premature birth with insufficient development in utero poses crucial risks to the lifelong health of the offspring [14].

Third, the association between exposure and health risks is mediated by irregular epigenetic regulation. As observed in this study, maternal Cd exposure is associated with the hypermethylation of cg21010642, which subsequently affects SGA. Cg21010642 is located within the open sea region of the Jumonji and AT-rich interaction domain containing 2 (*JARID2*) gene, which plays a critical role in embryonic development [34]. JARID2 acts as a transcription repressor that recruits polycomb repressive complex 2 (PRC2) protein to activate trimethylation at lysine 27 in histone H3 (H3K27me3). However, the downregulation of *JARID2* repressed H3K27me3 modification, leading to the delayed repression of a transcription factor named NANOG, which is involved in embryonic development [34,35]. The persistent expression of *NANOG* consequently represses *β-catenin* [34], which may interrupt early development through the abnormal regulation of the Wnt/β-catenin signaling pathway. Zhang et al. [36,37] reported that the reduced expression of *Wnt2* and *β-catenin* might be attributable to preeclampsia, the outcome of which includes PTB [38]. Considering that DNA methylation is inversely associated with gene expression, the hypermethylation of cg21010642 may play a role as an initiation factor for successive mechanisms. To the best of our knowledge, cg21010642 has not been reported to be associated with PTB or SGA. Still, several other CpG sites adjacent to *JARID2* are found to be hypermethylated in premature or fetal-growth-restricted babies [39]. Therefore, it is expected that cg21010642, along with other CpG sites, participates in *JARID2* repression and contributes to prematurity. In other words, cg21010642 may play a role as a predictive marker for maternal Cd exposure and the possibility of preterm birth.

This study was aimed at investigating the causal and mediating effects of the three factors of the exposome. However, there are several limitations to this study. First, some of the association studies had low statistical power, presumably because of the sample size. Although the number of subjects is thought to be sufficient, the majority had normal ranges of exposure levels or birth outcomes. However, this skewness is a common characteristic of exposome studies that focus on the normal population, rather than on specific individuals who are highly exposed because of accidents [40] or their occupations [41,42,43]. Second, we were unable to utilize RNA samples to verify the expression patterns of *JARID2* according to the differential methylation of cg21010642. Although integrated omics analyses [42,43] would have ensured the validity of the observation, obtaining eligible materials, such as RNA, from subjects who were recruited over a decade ago was difficult. The amount and state of the biological samples collected was limited, as is usual in cohort studies. RNAs are structurally unstable, and thus cohort studies are often hampered by having to extract and maintain the proper amount of RNA for downstream applications [44,45]. Furthermore, it is difficult to obtain a proper quantity of samples from subjects, especially from infants or children in the birth cohort. The procurement of suitable samples is a crucial hurdle in the progression of exposome research.

Nevertheless, this study advances exposome research by integrating three factors of the exposome and identifying their systematic association. For future directions, ensuring a proper sample size and efficiency and obtaining diverse sample types, such as DNA, RNA, and chromatin, would enable multidisciplinary exposome interpretation [46,47].

## Figures and Tables

**Table 1:** Demographic description of subjects after imputation and filtration (mean ± standard deviation).

Newborns (*n* = 367)		Mothers (of 367 Newborns)	
Boys	186	Age (year)	30.4 ± 3.6
Girls	181	BMI (kg/m^2^)	22.9 ± 3.1
Gestational age (day)	275.4 ± 8.2	Smoker (yes/no)	38/329
Preterm birth (case/control)	5/362	Parity (0/>0)	209/158
Hg (μg/L)	5.9 ± 2.8	Hg (μg/L)	3.6 ± 1.9
Pb (μg/L)	10 ± 4.2	Pb (μg/L)	13.7 ± 5.7
Cd (μg/L)	0.7 ± 0.2	Cd (μg/L)	1.6 ± 0.4

**Table 2:** Association between maternal and prenatal exposure and between exposure and gestational age.

		F-Statistics (*p*)	Pearson’s Coefficients
**Maternal–Prenatal exposure**			
Hg		2.277 × 10^−67^ ***	0.750
Pb		3.773 × 10^−25^ ***	0.505
Cd		0.013 *	0.130
**Exposure–Gestational age**			
Maternal	Hg	0.924	0.005
	Pb	0.199	−0.067
	Cd	0.303	−0.054
Prenatal	Hg	0.542	0.032
	Pb	0.170	−0.072
	Cd	0.983	−0.001

(* *p* < 0.05; *** *p* < 0.001).

**Table 3:** Differential methylation patterns of cg21010642 and its correlation with maternal Cd levels and gestational age.

	Differential Methylation (β)		Linear Regression	
	*p*-Value	*q*-Value	*p*-Value	Correlation
Maternal Cd	8.6 × 10^−6^	0.14	1.07 × 10^−5^	0.2275
Gestational age	5.4 × 10^−10^	2.7 × 10^−7^	6.4 × 10^−10^	−0.3154

## Data Availability

The data presented in this study are available on request from the corresponding author.
